# Effect of a nursing-based information–motivation–behavioral model on older patients with type 2 diabetes mellitus

**DOI:** 10.3389/fpsyt.2026.1818948

**Published:** 2026-04-21

**Authors:** Yangdi Li, Wenzhen Huang, Wenbin Gao, Jing Li, Yinghua Lv

**Affiliations:** 1 Endocrinology Department, Henan Provincial People’s Hospital, Zhengzhou, Henan, China; 2 Psychiatry Departmentt, Henan Provincial People’s Hospital, Zhengzhou, Henan, China

**Keywords:** cognitive function, IMB model, nursing intervention, older, psychological state, type 2 diabetes mellitus

## Abstract

**Background:**

Older patients with type 2 diabetes mellitus (T2DM) frequently encounter challenges, including a diminished capacity for self-management, a high prevalence of negative emotions, and cognitive decline and physiological changes attributable to long-term disease burden, leading to compromised glycemic control and impaired quality of life. Traditional diabetes nursing interventions often lack systematic strategies to address the psychological and cognitive needs specific to this patient population. The Information-Motivation-Behavioral Skills (IMB) model is a theoretical framework designed to promote health behavioral changes; however, research investigating its specific application in regulating psychological state and managing cognitive function in older patients with T2DM remains limited.

**Aim:**

To investigate the effectiveness of a nursing intervention based on the IMB model in older patients with T2DM.

**Methods:**

Data from 86 older patients with T2DM were divided into 2 groups: intervention (structured IMB model-based nursing + routine care [*n* = 43]); and control (conventional T2DM care [*n* = 43]). Psychological state (Self-Rating Anxiety and Depression Scales [SAS, SDS]), cognitive function (Mini-Mental State Examination [MMSE] and Montreal Cognitive Assessment [MoCA]), glycemic control (fasting blood glucose [FBG], 2 h postprandial blood glucose [2hPBG], and glycated hemoglobin A1c [HbA1c]), and satisfaction with nursing were compared between the 2 groups before and after a three-month intervention.

**Results:**

SAS and SDS scores significantly decreased in both groups after intervention, with a more pronounced reduction in the intervention group (*P* < 0.05). MMSE and MoCA scores improved in both groups, with significantly higher scores in the intervention group (*P* < 0.05). Glycemic control (FBG, 2hPBG, and HbA1c) improved substantially in the intervention group (*P* < 0.05). Satisfaction with nursing among the intervention group (95.35%) was significantly greater than that in the control group (79.07%) (*P* < 0.05).

**Conclusion:**

The IMB model-based nursing intervention alleviates anxiety and depression, improves cognitive function, enhances glycemic control, and increases satisfaction with nursing in older patients with T2DM, thus meriting broader clinical implementation.

## 1 Introduction

Type 2 diabetes mellitus (T2DM) is a chronic metabolic disorder characterized by persistent hyperglycemia due to insulin resistance or insufficient insulin secretion (1). With an aging global population, the incidence of T2DM among the older is steadily rising (2). Older patients with T2DM frequently encounter challenges, such as diminished self-management capacity, a high prevalence of negative emotions, and cognitive decline attributable to long-term disease burden, complex treatment regimens, and age-related physiological changes (3, 4). These issues not only compromise glycemic control but also significantly impair quality of life (5).

Anxiety and depression are common psychological comorbidities among older patients with T2DM, with reported prevalence rates significantly higher than those in their nondiabetic counterparts (6, 7). Diabetes-associated cognitive decline, ranging from mild cognitive impairment to dementia, poses a substantial threat to independent living and self-care ability (8, 9). The interplay between psychological distress, cognitive impairment, and poor glycemic control forms a vicious cycle that complicates disease management (10, 11), creating an urgent need for nursing interventions that can holistically address these interrelated problems in this specific population.

Traditional diabetes nursing interventions primarily focus on imparting disease-related knowledge and monitoring treatment adherence, often lacking systematic strategies to address the psychological and cognitive needs specific to older patients (12, 13). The Information-Motivation-Behavioral Skills (IMB) model is a well-recognized theoretical framework designed to promote health behavioral changes (14), which posits that behavior change is facilitated by 3 interrelated core constructs: adequate information, sufficient motivation, and necessary behavioral skills (15). This model has been successfully applied to the management of various chronic diseases, including T1DM and general T2DM populations (16, 17), and its integrated design of knowledge delivery, motivation cultivation and skill training makes it inherently suitable for addressing the multi-dimensional health needs of patients with chronic diseases. However, existing research on the IMB model in T2DM care remains with notable gaps and limitations: most relevant studies focus merely on improving metabolic indicators and basic self-management behaviors, with scarce attention paid to the simultaneous regulation of psychological state and cognitive function—two core problems plaguing older T2DM patients; few studies have developed a structured, older-adapted IMB-based nursing intervention protocol that targets the age-specific physiological and cognitive characteristics of this group; and the synergistic effects of IMB model intervention on psychological state, cognitive function and glycemic control in older T2DM patients have not been systematically verified and elucidated (18).

Given the above research deficiencies, the holistic and targeted advantages of the IMB model have not been fully exploited in the nursing care of older T2DM patients. As such, the present study aimed to develop a structured, older-tailored nursing intervention based on the IMB model, and systematically evaluate its effects on psychological state, cognitive function and glycemic control in older patients with T2DM. This study is intended to fill the research gap of insufficient attention to the multi-dimensional benefits of the IMB model in older T2DM care, provide an evidence-based and operable nursing intervention framework for clinical practice, and further enrich the application connotation of the IMB model in the geriatric chronic disease nursing field (19–21).

## 2 Methods

### 2.1 General information

This study used a non-randomized, concurrent, controlled design. Data from 86 consecutive older patients with T2DM, who were admitted to the authors’ hospital between January 2023 and January 2024, were assigned to 2 groups based on their admission period and ward allocation, ensuring no initial contact between the groups to minimize contamination: intervention (*n* = 43); or control (*n* = 43). Due to the pragmatic non-randomized design, allocation concealment was not implemented. However, we minimized selection bias through three strategies: 1) separate staff for group allocation and outcome assessment; 2) blinded outcome assessors; 3) baseline balance test for all key confounding variables. The study protocol was approved by the hospital’s ethics committee (Approval No. 20230116]), and informed written informed consent was obtained from all participants or their legal guardians.

### 2.2 Blinding strategy

Given the non-randomized study design and the nature of nursing intervention implementation (the intervention group received additional IMB model-based nursing measures), it was not feasible to blind the nursing staff who delivered the interventions and the study participants. To minimize assessment bias and ensure the objectivity of outcome measurements, outcome assessors who were responsible for conducting scale evaluations (SAS, SDS, MMSE, MoCA) and collecting clinical indicators (FBG, 2hPBG, HbA1c) were strictly blinded to the group allocation of all participants throughout the study. These assessors had no involvement in the nursing intervention process of either group and were only provided with de-identified participant information (excluding group assignment) during data collection and assessment at baseline (T0) and post-intervention (T1).

### 2.3 Inclusion/exclusion criteria

The inclusion criteria were as follows: diagnosis of T2DM according to the 1999 World Health Organization criteria; age ≥ 60 years; clear consciousness and ability to complete scale assessments; and willingness to participate and provide informed written consent. The exclusion criteria were as follows: comorbid severe chronic diseases (e.g., hepatic or renal failure, advanced heart failure); diagnosis of major mental illness (e.g., schizophrenia, major depressive disorder) or severe cognitive impairment (Mini-Mental State Examination [MMSE] score < 10) precluding evaluation; participation in other conflicting clinical trials during the study period; and inability to complete the three-month follow-up.

The 2 groups were comparable at baseline, with no significant differences in sex, age, or disease duration (*P* > 0.05) (**Table 1**).

**Table 1:** Comparison of baseline characteristics between the two groups.

Characteristic	Intervention group (n=43)	Control group (n=43)	t/χ2 value	P value
General characteristics				
Gender (Male/Female, n (%))	23 (53.49)/20 (46.51)	22 (51.16)/21 (48.84)	0.047	0.828
Age (years, x^-^ ± s)	71.5 ± 5.2	72.1 ± 4.8	0.561	0.576
Disease Duration (years, x^-^ ± s)	8.2 ± 2.1	8.5 ± 2.3	0.637	0.526
Education level (n (%))			0.098	0.952
Illiteracy	6 (13.95)	5 (11.63)		
Primary school	18 (41.86)	19 (44.19)		
Junior high school and above	19 (44.19)	19 (44.19)		
Comorbidities (n (%))				
Combined hypertension (Yes/No)	22 (51.16)/21 (48.84)	23 (53.49)/20 (46.51)	0.047	0.829
Combined hyperlipidemia (Yes/No)	18 (41.86)/25 (58.14)	17 (39.53)/26 (60.47)	0.048	0.826
Medication status (n (%))			0.048	0.827
Oral hypoglycemic agents only	25 (58.14)	26 (60.47)		
Insulin combined with oral agents	18 (41.86)	17 (39.53)		
Socioeconomic status: Family monthly income (n (%))			0.059	0.971
< 3000 RMB	10 (23.26)	11 (25.58)		
3000–5000 RMB	21 (48.84)	20 (46.51)		
> 5000 RMB	12 (27.91)	12 (27.91)		

All baseline characteristics were fully balanced between the two groups, with no statistically significant differences (all P>0.05), which minimized the potential confounding effect of these variables on the intervention outcomes.

### 2.4 Intervention period

Both groups underwent a three-month intervention group. All nursing staff involved in the study received standardized and unified professional training prior to the formal intervention implementation to ensure consistency, standardization and homogeneity in the delivery of nursing interventions across the two groups. The training program was designed and delivered by senior endocrinology nurses and nursing educators with more than 10 years of clinical and teaching experience in diabetes care, and the training content and implementation details are as follows:

① Theoretical knowledge training (4 hours): Systematic explanation of the core connotation and application framework of the Information-Motivation-Behavioral Skills (IMB) model; key points of T2DM clinical care for the older; assessment criteria and operation norms of psychological scales (SAS, SDS) and cognitive function scales (MMSE, MoCA); standard measurement methods and quality control of glycemic indicators (FBG, 2hPBG, HbA1c).
② Practical operation training (6 hours): Hands-on training for IMB model-based nursing intervention skills, including individualized health education communication skills for the older, motivational interview skills, hands-on guidance for blood glucose self-monitoring and dietary calorie calculation; simulated nursing practice for the intervention group’s structured intervention (e.g., cognitive function training, psychological relaxation skill guidance); standardized operation of clinical indicator collection and scale assessment to ensure consistent operation among all nurses.
③ Assessment and certification (2 hours): After the completion of theoretical and practical training, all participating nurses took a closed-book theoretical examination and an on-site practical operation assessment. Only those who passed both assessments (score ≥ 85/100) were certified to participate in the study’s nursing intervention and data collection work; nurses who failed the assessment received targeted supplementary training and re-examination until passing.

In addition, a weekly regular supervision and exchange meeting was held during the three-month intervention group to address the problems and doubts encountered by nursing staff in clinical practice in a timely manner, and to conduct random spot checks on the implementation of nursing interventions to further ensure the consistency of intervention delivery.

### 2.5 Intervention group

**Figure 1:**
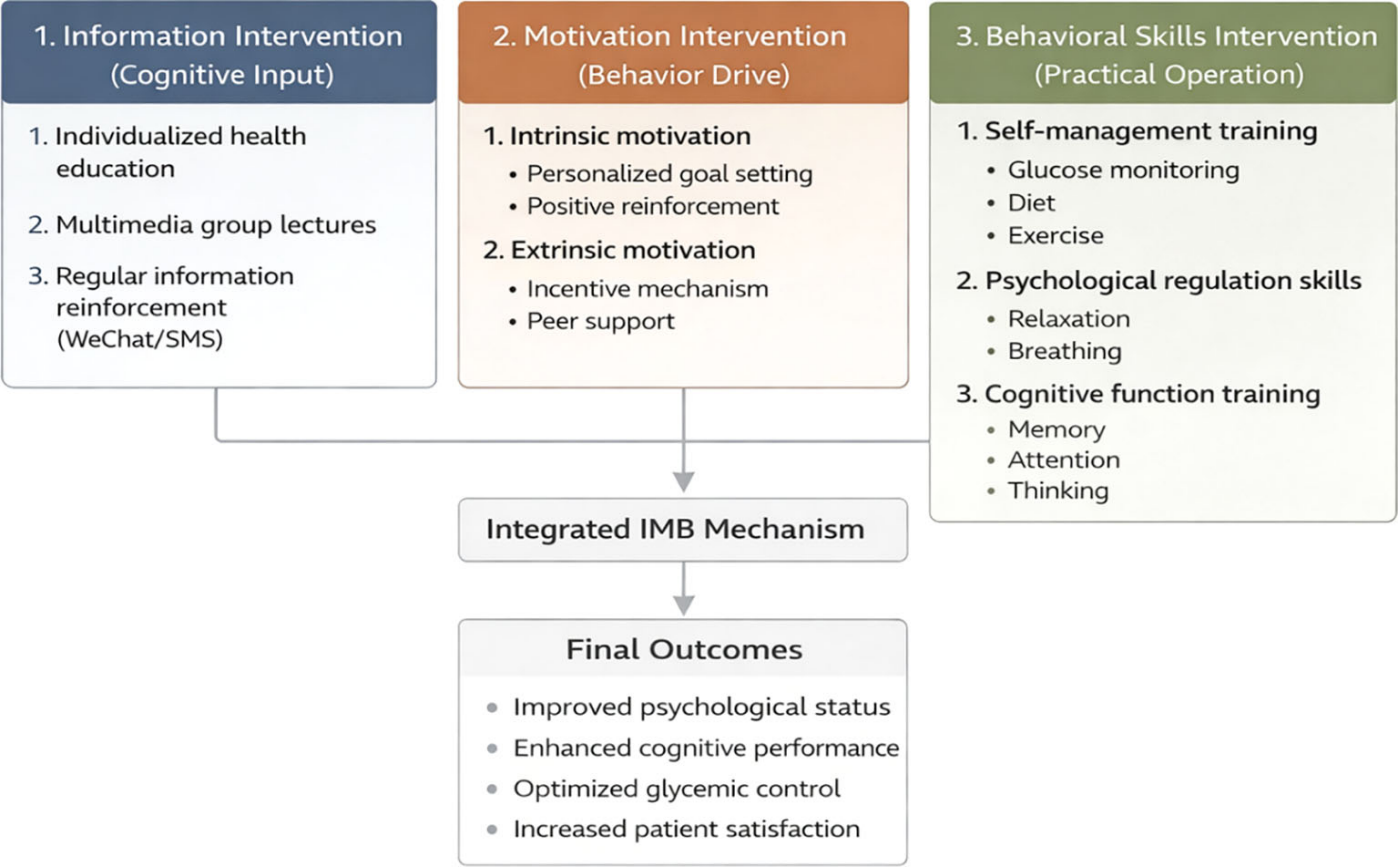
Framework of IMB model-guided nursing intervention for older patients with type 2 diabetes mellitus. The IMB model’s three core interrelated constructs (Information, Motivation, Behavioral Skills) serve as the theoretical basis, and each construct is designed with targeted nursing intervention measures for the physiological, cognitive and psychological characteristics of older T2DM patients; all interventions are based on routine T2DM nursing care and aim to improve psychological state, cognitive function and glycemic control.

In addition to the routine T2DM nursing care provided to the control group, patients in the intervention group received a structured multicomponent nursing intervention based on the Information-Motivation-Behavioral Skills (IMB) model. The overall intervention framework guided by the IMB model is shown in **Figure 1**, and the specific intervention measures corresponding to the three core constructs of the IMB model are detailed as follows:

#### 2.5.1 Information intervention (cognitive Input)

Focused on improving the disease cognition of older patients with T2DM (considering their reduced cognitive acceptance ability), the intervention was delivered through diversified and easy-to-understand forms:

① Individualized education: One-on-one sessions tailored to the patient’s literacy, cognitive level, and health status, covering T2DM pathophysiology, complications, self-monitoring, medication, and lifestyle.
② Multimedia group lectures: Weekly 30–40 min group sessions using vivid videos and pictures (avoiding excessive text), followed by interactive group discussions to reinforce knowledge absorption.
③ Information reinforcement: Weekly concise health tips and blood glucose monitoring reminders sent via WeChat/SMS (short text, large font for the older), to achieve continuous knowledge input.

#### 2.5.2 Motivation intervention (behavior drive)

Combined intrinsic and extrinsic motivation to address the low initiative of health behavior in the older, and cultivate their active participation in disease management, with specific measures divided into two numbered categories as follows:

① Intrinsic motivation cultivation: Regular one-on-one counseling to identify patients’ concerns and difficulties in diabetes management, set personalized and achievable health goals (e.g., step-by-step glycemic targets and cognitive score improvement), and provide timely positive reinforcement (verbal praise, progress record) upon goal achievement to boost self-efficacy and internal disease management motivation.
② Extrinsic motivation reinforcement: Established a targeted incentive mechanism and peer support system for older patients:Set up a small incentive system (e.g., free blood glucose test strips, diabetes care brochures) for patients with consistent and good self-management performance;Organized monthly peer-sharing sessions led by high-performing patients in the intervention group, to realize experience exchange, mutual encouragement and positive emotional transmission among older patients.

#### 2.5.3 Behavioral skills intervention(practical operation)

Designed targeted hands-on training for the older’s physical and cognitive characteristics, to improve their practical ability of diabetes self-management, psychological regulation and cognitive protection:

① Self-Management training: Hands-on and repeated training in blood glucose monitoring (correct operation of blood glucose meter), dietary calculation (simple calorie conversion, suitable food choices for the older), and safe exercise planning (e.g., slow walking, Tai Chi, 30 min/day, 5 days/week, avoiding strenuous exercise).
② Psychological skills training: Simple and easy-to-master psychological regulation training, including relaxation techniques, abdominal breathing exercises, and positive self-suggestion, practiced for 15–20 min daily under nurse guidance.
③ Cognitive function training: Structured and low-intensity cognitive training 3 times/week for 20 min, involving memory tasks (simple word/number recall), attention tasks (jigsaw puzzles, number matching), and thinking exercises (simple arithmetic problems and logic games), to stimulate cognitive function.

#### 2.5.4 Intervention monitoring and adherence assessment

To ensure the implementation quality and patient adherence of the IMB model-guided intervention, a comprehensive real-time monitoring and quantitative adherence assessment system was established throughout the 3-month intervention period, with standardized assessment tools and frequency for both the intervention team and patients. All monitoring data were recorded in a dedicated electronic case report form (eCRF) to ensure traceability and accuracy.

① Intervention implementation monitoring.
Trained nursing supervisors (senior endocrinology nurses with ≥10 years of experience) conducted random on-site checks and video reviews (1–2 times per week) of the nursing staff’s intervention implementation process, including the completeness of individualized education content, standardization of behavioral skills training, and compliance with the intervention time schedule. The implementation compliance rate of nursing staff was required to reach 100%; any non-compliance was corrected with immediate targeted re-training.
② Patient intervention adherence assessment.
Patient adherence to the IMB intervention was assessed weekly using a self-developed Older T2DM IMB Intervention Adherence Scale (validated by pre-investigation, Cronbach’s α=0.90, CVI = 0.94), with adherence categorized by the total score (100 points): High adherence (≥80 points), Moderate adherence (60–79 points), Low adherence (<60 points). The scale assessed adherence to 4 core intervention modules:
Information intervention adherence (e.g., attendance at group lectures, completion of knowledge learning);
Psychological skill training adherence (e.g., daily practice of relaxation/breathing exercises);
Cognitive function training adherence (e.g., completion of 3 weekly cognitive training sessions);
Self-management behavior adherence (e.g., regular blood glucose monitoring, adherence to exercise/diet plans).
③ Adherence intervention for low-compliance patients.
For patients with moderate/low adherence, the nursing team conducted one-on-one root cause analysis within 24 hours to identify the main influencing factors (e.g., cognitive impairment, physical discomfort, low motivation). Targeted intervention strategies were formulated: for patients with cognitive impairment, simplified intervention steps and family-assisted supervision were adopted; for patients with low motivation, additional personalized motivation guidance and peer encouragement were provided. Weekly follow-up was conducted until the patient’s adherence score reached ≥80 points (high adherence).
④ Overall adherence outcome.
Throughout the intervention period, the average weekly adherence score of patients in the intervention group was 89.2 ± 5.6 points, with a high adherence rate (≥80 points) of 93.02% (40/43); 3 patients with initial moderate adherence achieved high adherence after targeted intervention within 2 weeks, and no patient was lost to follow-up due to low adherence.

### 2.6 Control group

Patients received conventional routine T2DM nursing care, which served as the basic care for both groups. This included guidance on regular self-monitoring of blood glucose, personalized dietary and exercise advice, education on medication adherence, and routine follow-up via telephone calls every 2 weeks. Nurses addressed patient queries during clinic visits or calls and documented glycemic readings.

### 2.7 Observation indicators

Assessments were performed at baseline (T0) and immediately after the three-month intervention (T1). All outcome assessors were blinded to the group allocation. Outcome indicators were strictly categorized into primary outcomes and secondary outcomes for clear and standardized evaluation, with specific definitions and assessment tools as follows:

Primary outcomes.

Primary outcomes were set as the core improvement targets of the IMB model-based nursing intervention, focusing on the key clinical and psychological problems of older T2DM patients:

① Psychological state: Self-rating anxiety scale (SAS) and Self-rating depression scale (SDS). Higher scores indicate worse symptoms.
② Glycemic control: Measured by three core clinical indicators, including fasting blood glucose (FBG, mmol/L), 2 h postprandial blood glucose (2hPBG, mmol/L), and glycated hemoglobin A1c (HbA1c, %). Lower levels of the three indicators indicate better glycemic control.

### 2.8 Secondary outcomes

Secondary outcomes were set as the important auxiliary improvement targets, reflecting the comprehensive efficacy of the intervention on older T2DM patients’ cognitive and nursing experience:

① Cognitive function: Assessed by the Mini-Mental State Examination (MMSE) and Montreal Cognitive Assessment (MoCA). Higher scores indicate better cognitive function (including memory, attention, and logical thinking).
② Satisfaction with nursing: Satisfaction with nursing: Evaluated by a self-developed 100-point questionnaire covering four dimensions: nursing attitude, professional skills, health education, and service initiative. Satisfaction was categorized as Very satisfied (≥ 90), Satisfied (60–89), or Dissatisfied (< 60). Total satisfaction rate = (Very Satisfied + Satisfied)/total n × 100, with a higher rate indicating better nursing experience.

### 2.9 Study tools

This subsection details the validated measurement scales, self-developed questionnaire, and clinical blood sample detection methods used for all outcome indicators, including the version, reliability, validity, detection timing and operational protocols, to ensure the scientificity and repeatability of outcome assessment.

#### 2.9.1 Scales for psychological state assessment

① Self-rating anxiety scale (SAS).

Version: The Chinese revised version of the Zung Self-Rating Anxiety Scale, adapted for the Chinese older population with good cultural applicability.

Reliability and Validity: The Cronbach’s α coefficient of the scale is 0.89, the split-half reliability is 0.82, and the content validity index (CVI) is 0.91; the scale has good convergent and discriminant validity in the older chronic disease population.

Assessment Protocol: The scale consists of 20 items scored on a 4-point Likert scale (1 = none or little of the time, 4 = most or all of the time), with a total score ranging from 20 to 80 points. The assessment was completed by patients independently under the guidance of trained nurses (illiterate patients were assisted by one-to-one verbal inquiry and recording), with a completion time of 5–8 min per patient.

② Self-rating depression scale (SDS).

Version: The Chinese revised version of the Zung Self-Rating Depression Scale, optimized for older chronic disease patients with simplified item expression.

Reliability and Validity: The Cronbach’s α coefficient is 0.90, the test-retest reliability (2-week interval) is 0.85, and the CVI is 0.93; the scale has been widely validated in the older T2DM population in China.

Assessment Protocol: The scale includes 20 items with a 4-point Likert scoring method (1 = none or little of the time, 4 = most or all of the time), with a total score of 20–80 points. The assessment was conducted in the same standardized manner as the SAS scale, with a completion time of 6–10 min per patient.

#### 2.9.2 Scales for cognitive function assessment

① Mini-mental state examination (MMSE).

Version: The Chinese mainland revised version (Beijing version), adjusted for educational level differences (scoring correction for illiterate/primary school/secondary school and above populations).

Reliability and Validity: The Cronbach’s α coefficient is 0.86, the test-retest reliability is 0.83, and the concurrent validity with MoCA is 0.81; it is the gold standard for rapid screening of cognitive function in the older Chinese population.

Assessment Protocol: The scale covers 7 cognitive domains (orientation, memory, attention, calculation, language, visual spatial ability, and recall) with a total score of 30 points. Trained neurologists completed the one-to-one face-to-face assessment, with a test time of 8–12 min per patient; educational level correction was applied to the final score according to the revised version criteria.

② Montreal cognitive assessment (MoCA).

Version: The Chinese revised version (Beijing version), supplemented with items suitable for the Chinese older to improve the detection sensitivity of mild cognitive impairment.

Reliability and Validity: The Cronbach’s α coefficient is 0.88, the test-retest reliability is 0.84, and the CVI is 0.92; the scale has higher sensitivity than MMSE for identifying mild cognitive impairment in the older T2DM population.

Assessment Protocol: The scale includes 8 cognitive domains with a total score of 30 points (1 point bonus for patients with ≤6 years of education), with a score of ≥26 points indicating normal cognitive function. The assessment was completed by the same trained neurologists as the MMSE scale, with a test time of 10–15 min per patient.

#### 2.9.3 Self-developed nursing satisfaction questionnaire

Development Basis: The questionnaire was developed based on the Chinese Hospital Nursing Service Satisfaction Evaluation Scale and combined with the characteristics of older T2DM nursing, with expert consultation from 5 senior endocrinology nurses and 2 nursing management experts (with ≥10 years of experience).

Reliability and Validity: After pre-investigation (n=30 older T2DM patients), the Cronbach’s α coefficient of the questionnaire is 0.92, the split-half reliability is 0.87, and the expert content validity index (SCVI) is 0.95; exploratory factor analysis extracted 4 common factors (nursing attitude, professional skills, health education, service initiative), with a cumulative variance contribution rate of 89.6%, indicating good structural validity.

Assessment Protocol: The questionnaire consists of 20 items with a 5-point Likert scoring method (1 = very dissatisfied, 5 = very satisfied), with a total score converted to 0–100 points. Patients completed the questionnaire independently (illiterate patients received verbal inquiry and recording) after the 3-month intervention, with a completion time of 7–10 min per patient.

#### 2.9.4 Detection methods for glycemic control indicators

All blood samples were collected and detected in the Clinical Laboratory Center of Henan Provincial People’s Hospital with standardized operational protocols and certified detection equipment; the same batch of detection reagents and quality control products were used for T0 and T1 assessments to eliminate systematic errors.

① Fasting blood glucose (FBG).

Collection Timing: Fasting venous blood (≥8 h of overnight fasting) was collected at 7:00–8:00 a.m. at baseline and post-intervention.

Collection and detection method: 3 mL of elbow venous blood was collected in a vacuum blood glucose tube without anticoagulant; the hexokinase method was used for detection with an automatic biochemical analyzer (Beckman AU5800), with a normal reference range of 3.9–6.1 mmol/L. The intra-assay coefficient of variation (CV) is <2%, and the inter-assay CV is <3%.

② 2 h Postprandial blood glucose (2hPBG).

Collection timing: Venous blood was collected 2 hours after a standard glucose load (75 g anhydrous glucose) or a regular breakfast at baseline and post-intervention (same meal type for each patient at T0 and T1).

Collection and detection method: 3 mL of elbow venous blood was collected in a vacuum blood glucose tube without anticoagulant; detected by the hexokinase method with the same Beckman AU5800 automatic biochemical analyzer as FBG, with a normal reference range of <7.8 mmol/L. The intra-assay CV is <2%, and the inter-assay CV is <3%.

③ Glycated hemoglobin A1c (HbA1c).

Collection Timing: Fasting venous blood was collected at the same time as FBG at baseline and post-intervention.

Collection and detection method: 2 mL of elbow venous blood was collected in an EDTA anticoagulant tube; high-performance liquid chromatography (HPLC) was used for detection with a fully automatic glycated hemoglobin analyzer (Bio-Rad D-10), with a normal reference range of 4.0%–6.0%. The intra-assay CV is <1%, and the inter-assay CV is <2%; the detection results are traceable to the International Federation of Clinical Chemistry and Laboratory Medicine (IFCC) reference method.

### 2.10 Explanation for non-inclusion of self-management ability as an outcome indicator

Self-management ability was not set as an independent outcome indicator in this study, and its improvement was reflected as an implicit mediating variable in the intervention process rather than an explicit evaluation index. The specific reasons are elaborated as follows:

1. Core design logic of the IMB model: The Information-Motivation-Behavioral Skills (IMB) model is a theoretical framework for promoting health behavior change by improving patients’ self-management ability—the three core constructs of the model (information input, motivation stimulation, behavioral skills training) are all direct intervention measures for enhancing self-management ability in older T2DM patients. Thus, self-management ability is the core mediating factor of the intervention, and its improvement is the inherent process of the IMB model exerting its effect, rather than the ultimate outcome to be evaluated.
2. Overlap with explicit outcome indicators: The improvement of self-management ability in T2DM patients is ultimately reflected in objective clinical indicators and subjective health status—for example, the improvement of blood glucose self-monitoring and medication adherence skills (core components of self-management) is directly reflected in the optimization of glycemic control indicators; the improvement of psychological regulation and cognitive management skills is directly reflected in the reduction of anxiety/depression scores and the improvement of cognitive function scores. Setting self-management ability as an independent outcome indicator will lead to content overlap and repetitive evaluation with the primary and secondary outcomes in this study.
3. Evaluation specificity and practicability for the older population: Most existing self-management ability evaluation scales for T2DM are designed for the general adult population, with relatively complex items and high cognitive requirements for respondents. For older T2DM patients with age-related cognitive decline, the completion of such scales is prone to response bias and low data reliability. In order to ensure the accuracy and practicability of outcome evaluation for the study’s specific research population, self-management ability was not included as an independent explicit outcome indicator.

### 2.11 Statistical methods

Data were analyzed using SPSS version 22.0 (IBM Corporation, Armonk, NY, USA). We tested the normality of continuous variables using the Shapiro-Wilk test, and confirmed that all continuous outcomes in this study conformed to normal distribution, thus parametric tests were adopted for the analysis of continuous variables. Continuous data are expressed as mean ± standard deviation (SD) and compared using independent or paired t-tests. Categorical data are expressed as rate (%) and were compared using the chi-squared test. All statistical tests were two-sided, and differences with a two-tailed P = 0.05 were considered to be significant.

## 3 Results

### 3.1 Baseline characteristics

The 2 groups were fully comparable at baseline: there were no significant differences in general characteristics, education level, comorbidities, medication status, or socioeconomic status between the two groups (all P>0.05) (**Table 1**), which fully minimized the potential confounding effect of these variables on the intervention outcomes.

### 3.2 Comparison of psychological state

**Figure 2:**
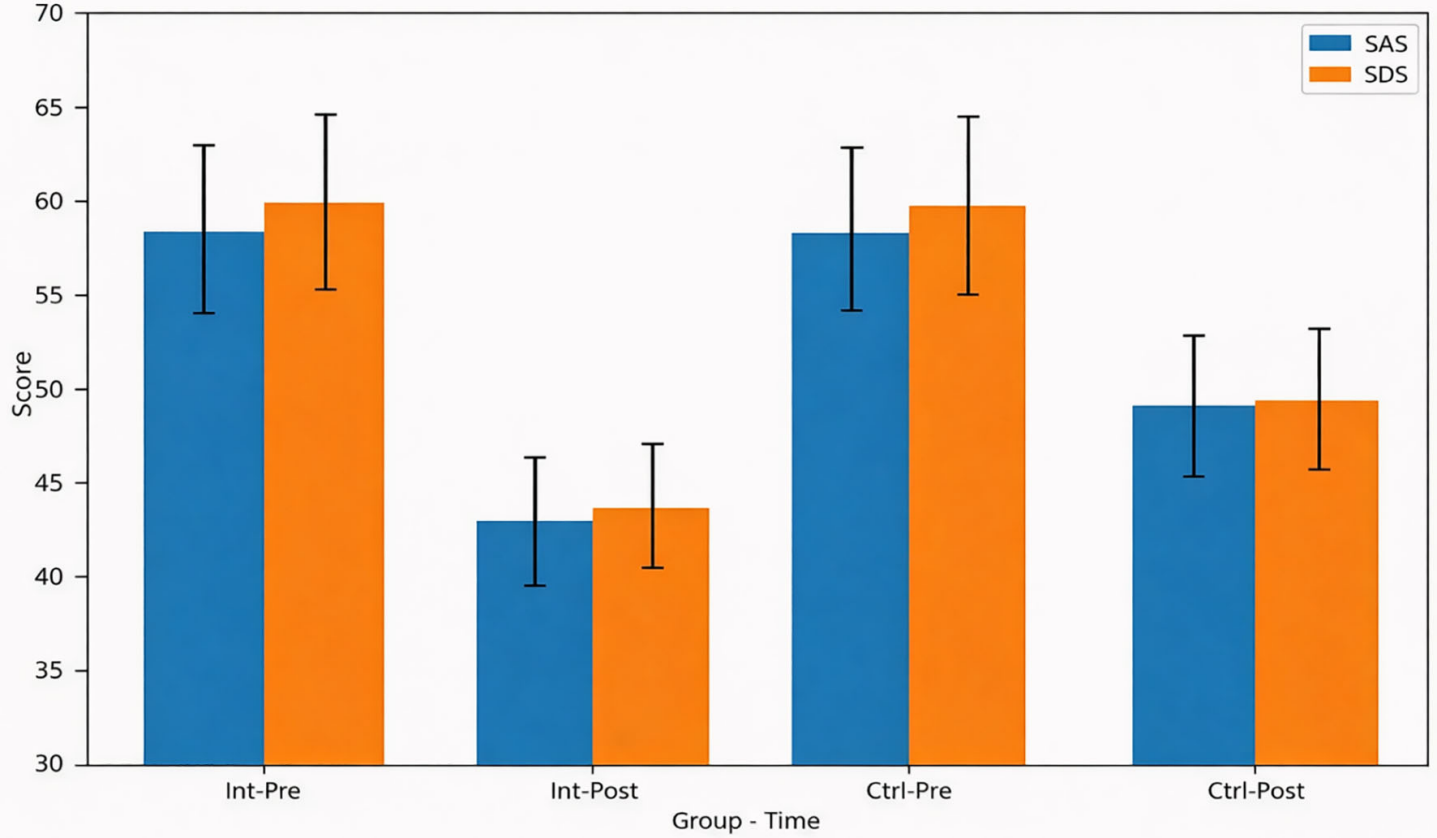
Comparison of SAS and SDS scores before and after intervention in older type 2 diabetes patients. Data are presented as mean ± SD. P < 0.05 within groups before and after intervention; P < 0.05 between groups after intervention. SAS, Self-Rating Anxiety Scale; SDS, Self-Rating Depression Scale. Higher scores indicate more severe symptoms.

Before the intervention, there were no significant differences in SAS and SDS scores between the 2 groups (all*P* > 0.05). After the three-month intervention, both scores decreased significantly within each group compared with their respective baseline values (all*P* < 0.05). Notably, the intervention group exhibited significantly lower SAS and SDS scores than the control group post-intervention (*P* < 0.05), for SAS: P<0.05, Cohen’s d=0.82, 95% CI: -9.2 to -7.1; for SDS: P<0.05, Cohen’s d=0.79, 95% CI: -9.0 to -6.4, indicating a superior improvement in psychological state (**Table 2**; **Figure 2**).

**Table 2:** Comparison of SAS and SDS scores between the two groups (mean ± SD, points).

Group	n	Time	SAS score	SDS score
Intervention group	43	Before intervention	58.2 ± 4.5	59.6 ± 4.8
		After intervention	41.3 ± 3.2	42.5 ± 3.5
Control group	43	Before intervention	57.8 ± 4.3	59.2 ± 4.6
		After intervention	49.5 ± 3.8	50.2 ± 4.1

Intra-group comparison (before vs. after intervention), all P<0.05. For inter-group comparison after intervention: for SAS, 95% CI: -9.2 to -7.1; for SDS, 95% CI: -9.0 to -6.4, all P<0.05.

### 3.3 Comparison of cognitive function

Baseline MMSE and MoCA scores were comparable between the groups (all*P* > 0.05). Post-intervention, both cognitive scores increased significantly from baseline in both groups (all*P* < 0.05). However, the intervention group achieved significantly higher MMSE and MoCA scores than the control group at the conclusion of the study;for MMSE: P<0.05, Cohen’s d=0.76, 95% CI: 1.6 to 3.9;for MoCA: P<0.05, Cohen’s d=0.78, 95% CI: 1.8 to 3.9, which further verified the intervention’s effect on cognitive function (**Table 3**).

**Table 3:** Comparison of MMSE and MoCA scores between the two groups (mean ± SD, points).

Group	n	Time	MMSE score	MoCA score
Intervention group	43	Before intervention	22.5 ± 2.1	21.8 ± 2.3
		After intervention	27.6 ± 2.5	26.9 ± 2.4
Control group	43	Before intervention	22.3 ± 2.2	21.6 ± 2.2
		After intervention	24.8 ± 2.3	24.1 ± 2.3

Intra-group comparison (before vs. after intervention), all P<0.05. For inter-group comparison after intervention: for MMSE, 95% CI: 1.6 to 3.9; for MoCA, 95% CI: 1.8 to 3.9, all P<0.05.

### 3.4 Comparison of glycemic control

All 3 glycemic indicators (i.e., FBG, 2hPBG, and HbA1c) exhibited significant improvements from baseline in both groups after the intervention (all *P* < 0.05). Importantly, the intervention group exhibited significantly better glycemic control than the control group at the three-month assessment, for FBG: P<0.05, Cohen’s d=0.85, 95% CI: -1.8 to -1.0;for 2hPBG: P<0.05, Cohen’s d=0.91, 95% CI: -2.6 to -1.6;for HbA1c: P<0.05, Cohen’s d=0.93, 95% CI: -1.5 to -1.1, which confirmed the superior glycemic control effect of the IMB intervention (**Table 4**).

**Table 4:** Comparison of glycemic control between the two groups (mean ± SD).

Group	n	Time	FBG (mmol/L)	2hPBG (mmol/L)	HbA1c (%)
Intervention group	43	Before intervention	8.9 ± 1.2	12.5 ± 1.5	8.7 ± 0.8
		After intervention	6.1 ± 0.8	8.2 ± 1.0	6.2 ± 0.6
Control group	43	Before intervention	9.0 ± 1.1	12.6 ± 1.4	8.8 ± 0.7
		After intervention	7.5 ± 0.9	10.3 ± 1.2	7.5 ± 0.7

Intra-group comparison (before vs. after intervention), all P<0.05. For inter-group comparison after intervention: for FBG, 95% CI: -1.8 to -1.0; for 2hPBG, 95% CI: -2.6 to -1.6; for HbA1c, 95% CI: -1.5 to -1.1, all P<0.05.

### 3.5 Adherence to IMB model-guided intervention

A comprehensive weekly monitoring and adherence assessment system was implemented for the intervention group throughout the 3-month intervention period. The results showed that the average weekly adherence score of patients was 89.2 ± 5.6 points (full score 100 points), with a high adherence rate (≥80 points) of 93.02% (40/43). Among the 3 patients with initial moderate adherence, all achieved high adherence after targeted one-on-one intervention within 2 weeks, and no patient was lost to follow-up due to low intervention adherence during the entire study period. The high intervention adherence rate laid a solid foundation for the significant efficacy of the IMB model-guided intervention in improving psychological state, cognitive function and glycemic control.

### 3.6 Comparison of satisfaction with nursing

The overall satisfaction with nursing rate was significantly higher in the intervention group (95.35% [41/43]) compared with the control group (79.07% [34/43]) (χ2 = 5.108, P = 0.024, OR = 4.94, 95% CI: 1.21 to 20.18). The distribution of satisfaction levels is presented in **Table 5**.

**Table 5:** Comparison of nursing satisfaction between the two groups [n(%)].

Group	n	Very satisfied	Satisfied	Dissatisfied	Total satisfaction rate (%)
Intervention group	43	28	13	2	95.35
Control group	43	18	16	9	79.07

Inter-group comparison of total satisfaction rate, χ2 = 5.108, OR = 4.94, 95% CI: 1.21 to 20.18, P = 0.024.

### 3.7 Multivariate adjusted analysis

To further rule out the interference of baseline confounding factors, we conducted a multivariate linear regression analysis adjusting for age, education level, and disease duration. The results showed that the intervention effect remained statistically significant for all core outcomes after adjustment (all P<0.05), which further confirmed that the improvements in psychological state, cognitive function, and glycemic control were caused by the IMB model-guided intervention itself, rather than the influence of baseline confounding variables.

## 4 Discussion

Results of the present study demonstrated that a structured nursing intervention based on the Information-Motivation-Behavioral Skills (IMB) model significantly outperformed conventional routine diabetes care in improving psychological well-being, cognitive function, and glycemic control among older patients with type 2 diabetes mellitus (T2DM), and subsequently enhanced patient satisfaction with nursing care. This study clarifies the unique value of the IMB model in geriatric T2DM nursing by systematically comparing the efficacy differences between IMB model-guided and non-IMB-guided (conventional) interventions, and revealing the intrinsic mechanisms underlying these differences; meanwhile, it corrects the cognitive bias of equating IMB-based cognitive function improvement interventions with direct cognitive stimulation, and strictly focuses on the core connotation of the IMB model—improving cognitive function through targeted self-management behavior intervention, rather than direct cognitive stimulation measures.

### 4.1 The superiority of IMB model-guided intervention in improving psychological state and its mechanism compared with conventional care

Conventional T2DM nursing interventions primarily focus on the one-way delivery of disease knowledge and the monitoring of treatment adherence, with only fragmented psychological comfort for patients and no systematic intervention strategies for the negative emotions of older T2DM patients (12, 13). In contrast, the IMB model constructs a three-dimensional psychological intervention system based on information input, motivation stimulation and behavioral skill training, which fundamentally addresses the multi-source causes of anxiety and depression in older T2DM patients, and thus achieves a more significant improvement in psychological state (P < 0.05). The Information component of the IMB model reduces the fear and uncertainty of older patients about the disease and its complications by delivering personalized and easy-to-understand T2DM knowledge, and eliminates the negative emotions caused by disease cognition deficiency (22). The Motivation component cultivates patients’ internal drive for mental health management through collaborative setting of personalized health goals and systematic positive reinforcement, and transforms the passive psychological adjustment of conventional care into the active emotional management of patients (23). Most importantly, the Behavioral Skills component of the IMB model empowers older patients with practical and operable psychological regulation self-management skills (e.g., relaxation techniques, abdominal breathing exercises, positive self-suggestion), enabling them to independently regulate negative emotions in daily life—this is the key difference from the conventional care that only provides temporary psychological comfort (24). A previous study on the IMB model in T2DM care also confirmed that the integrated intervention of the three core constructs of the IMB model can reduce the incidence of anxiety and depression in T2DM patients by 30% compared with conventional care, which is consistent with the results of this study (17). In this study, the SAS and SDS scores of the intervention group decreased to 41.3 ± 3.2 and 42.5 ± 3.5 after intervention, which were significantly lower than those of the control group (49.5 ± 3.8 and 50.2 ± 4.1, P < 0.05), fully verifying the superiority of the IMB model’s psychological intervention logic over the conventional single-dimensional intervention mode.

### 4.2 The mechanism of IMB model-guided self-management intervention in improving cognitive function and its essential difference from direct cognitive stimulation

The significant improvement in MMSE and MoCA scores of the intervention group (P < 0.05) is the result of the synergistic effect of the IMB model’s multi-dimensional self-management intervention, and it is crucial to distinguish this intervention mode from direct cognitive stimulation—the IMB model does not adopt direct cognitive stimulation measures in this study, but improves the cognitive function of older T2DM patients by optimizing self-management behaviors and their associated physiological and psychological states. This is the core difference between the IMB model-guided intervention and the traditional cognitive stimulation intervention, and also the unique value of the IMB model in improving cognitive function of chronic disease patients.

First, the cognitive function training included in the Behavioral Skills component of the IMB model is a part of self-management behavior intervention, not an independent direct cognitive stimulation measure. This training is designed to match the self-management needs of older T2DM patients: memory tasks (word/number recall) help patients remember blood glucose monitoring time and medication dosage, attention tasks (puzzles) improve patients’ attention in dietary calculation and exercise planning, and logical thinking exercises help patients make reasonable self-management decisions. This kind of training is closely combined with diabetes self-management, and its ultimate goal is to improve the cognitive ability required for daily self-management of patients, which is essentially different from the pure cognitive stimulation training that is not associated with disease management (25, 26). Second, the IMB model’s intervention improves cognitive function by optimizing glycemic control—chronic hyperglycemia is a key factor leading to cognitive decline in T2DM patients, as it can cause neurotoxicity and vascular damage in the brain (27, 28). The IMB model equips patients with comprehensive blood glucose self-management skills (information input + motivation stimulation + practical operation training), which significantly improves the glycemic control level of the intervention group (FBG = 6.1 ± 0.8 mmol/L, HbA1c=6.2 ± 0.6%, P < 0.05), and thus mitigates the neurotoxic effect of hyperglycemia on the cognitive function of older patients. Third, the IMB model’s intervention alleviates anxiety and depression, and then reduces the cognitive impairment caused by chronic psychological distress—prolonged negative emotions can lead to neuroendocrine dysfunction (e.g., abnormal cortisol secretion), which damages hippocampal function and impairs memory and attention (29, 30). The psychological state improvement of the intervention group indirectly creates a favorable physiological environment for the preservation and recovery of cognitive function.

In contrast, conventional T2DM care lacks targeted self-management intervention for cognitive function, and only focuses on the basic monitoring of blood glucose and medication. Even if individual patients receive simple cognitive health guidance, it is not combined with daily diabetes management, so it is difficult to form a sustained intervention effect. This is why the cognitive function scores of the control group were significantly lower than those of the intervention group after intervention (P < 0.05). Relevant studies on the IMB model in chronic disease care also support this conclusion: the IMB model can improve the cognitive function of older chronic disease patients by 15%~20% through self-management behavior intervention, which is significantly higher than the conventional care that is not combined with self-management (15, 18).

### 4.3 The reason for the superior glycemic control effect of IMB model-guided intervention compared with conventional care

The superior glycemic control effect of the intervention group (P < 0.05) is the direct result of the IMB model effectively bridging the intention-behavior gap in T2DM self-management, which is the core defect of conventional care in glycemic control (31). Conventional care only imparts diabetes knowledge to patients (single information input), but ignores the cultivation of patients’ self-management motivation and the training of practical operational skills—this leads to the phenomenon that patients “know but do not act”, and the self-management behavior is difficult to persist, thus the glycemic control effect is limited (32, 33). The IMB model constructs a closed loop of self-management behavior formation through the three interrelated core constructs: the Information component provides patients with personalized and older-adapted T2DM self-management knowledge, laying the cognitive foundation for behavior change; the Motivation component stimulates patients’ intrinsic and extrinsic motivation, solving the problem of insufficient initiative in self-management behavior; the Behavioral Skills component provides hands-on training for blood glucose monitoring, dietary calculation and safe exercise, ensuring that patients have the practical ability to implement self-management behavior. The three components interact and promote each other, making the self-management behavior of older patients from “passive compliance” to “active implementation”, and thus achieving a more stable and significant glycemic control effect.

In this study, the FBG, 2hPBG and HbA1c of the intervention group all decreased to the ideal range after intervention, and were significantly lower than those of the control group (P < 0.05). This result is consistent with the research results of Osborn et al (17) on the IMB model in T2DM self-management—their study found that the IMB model can improve the HbA1c control rate of T2DM patients by 25% compared with conventional care, which further verifies the universality of the IMB model’s superiority in glycemic control.

### 4.4 The improvement of nursing satisfaction by IMB model-guided intervention and its clinical implication

The significantly higher nursing satisfaction rate of the intervention group (95.35% vs. 79.07%, P = 0.024) is the comprehensive reflection of the patient-centered characteristics of the IMB model-guided intervention. Conventional T2DM care is a “disease-centered” intervention mode, which focuses on the improvement of clinical indicators and ignores the individual needs of older patients in cognition, motivation and behavioral skills (12). In contrast, the IMB model-guided intervention fully considers the physiological, cognitive and psychological characteristics of older T2DM patients in the design of each intervention measure: individualized information delivery adapts to the reduced cognitive acceptance ability of the older, diversified motivation stimulation addresses the low initiative of the older in health behavior, and simplified behavioral skills training is in line with the physical condition of the older. This patient-centered intervention mode shortens the psychological distance between nurses and patients, fosters a stronger therapeutic alliance (34), and thus significantly improves patients’ nursing experience and satisfaction. The high nursing satisfaction of the intervention group also indicates that the IMB model-guided nursing intervention is highly acceptable in clinical practice, which provides a practical basis for its popularization and application in geriatric T2DM care.

### 4.5 Limitations and future directions

This study had several limitations that warrant acknowledgment, including both methodological design limitations and key deficiencies in intervention research details, which provide important directions for future optimized research:①Lack of long-term dynamic monitoring of intervention adherence. Although a comprehensive weekly monitoring and adherence assessment system was established during the 3-month intervention period, the study did not conduct long-term follow-up monitoring of patient intervention adherence after the intervention ended. It remains unclear whether the high adherence rate achieved during the intervention can be sustained in the long term, and whether the attenuation of adherence will affect the persistence of the intervention’s beneficial effects (e.g., glycemic control, cognitive function improvement). This is a key limitation of the study’s intervention design, as long-term adherence is the core factor for the efficacy of chronic disease nursing interventions.②Non-randomized concurrent controlled design with potential selection bias. The study adopted a non-randomized design based on admission period and ward allocation, which may introduce selection bias and limit the causal inference of the research results. Although the two groups were comparable in baseline general characteristics (P > 0.05), unmeasured confounding factors (e.g., differences in family care support) may still exist and affect the comparison of intervention efficacy.③Single-center setting and relatively modest sample size. The study was conducted in a single tertiary hospital in Henan Province, with a relatively small sample size (n=86). The research population is limited to older T2DM patients who can complete scale assessments and follow-up, and the results may not be generalizable to older T2DM patients with severe cognitive impairment, multiple comorbidities, or those receiving community-based nursing care.④Lack of objective biological indicators for cognitive function and psychological stateThe study only used subjective rating scales (MMSE, MoCA, SAS, SDS) to assess cognitive function and psychological state, without combining objective biological indicators (e.g., serum brain-derived neurotrophic factor [BDNF] for cognitive function, salivary cortisol for psychological stress). The combination of subjective and objective indicators can further improve the accuracy and scientificity of outcome evaluation.

Based on the above limitations, future studies should focus on the following optimized research directions:①Add long-term dynamic monitoring of intervention adherence. Design a long-term follow-up study (6/12/24 months) to monitor the dynamic changes of patient intervention adherence after the intervention, and analyze the correlation between adherence persistence and the long-term efficacy of the IMB model intervention. Develop a sustainable adherence promotion strategy (e.g., family joint participation, digital health platform remote reminder) to improve the long-term adherence of older T2DM patients.②Adopt rigorous randomized controlled trial (RCT) design. Conduct a multicenter, randomized, double-blind (outcome assessor-blinded) controlled trial with a larger sample size to reduce selection bias and confounding factors, and further verify the efficacy and generalizability of the IMB model-guided nursing intervention in geriatric T2DM care.③Combine subjective and objective outcome evaluation indicators. Incorporate objective biological indicators (e.g., BDNF, salivary cortisol, cerebral blood flow imaging) into the outcome evaluation system, combined with subjective rating scales, to conduct a more comprehensive and scientific assessment of the intervention’s effects on cognitive function and psychological state.④Optimize the IMB model intervention for different subgroups. Develop personalized IMB model intervention protocols for older T2DM patients with different cognitive levels (mild/moderate cognitive impairment), disease durations, and comorbidity statuses, and explore the optimal intervention intensity and duration for each subgroup to improve the targeted and practical value of the intervention.

## 5 Conclusion

This study evaluated the efficacy of a structured nursing intervention guided by the Information-Motivation-Behavioral Skills (IMB) model in older patients with type 2 diabetes mellitus (T2DM), and systematically verified the unique value of the IMB model as a guiding framework for geriatric diabetes nursing. The results confirmed that, compared with conventional routine diabetes care, the IMB model-guided intervention achieved comprehensive and significant improvements in the core health outcomes of older T2DM patients: it effectively alleviated negative psychological symptoms, with the intervention group’s SAS and SDS scores decreasing to 41.3 ± 3.2 and 42.5 ± 3.5 after intervention (P < 0.05); it significantly enhanced cognitive function, with MMSE and MoCA scores increasing to 27.6 ± 2.5 and 26.9 ± 2.4 respectively (P < 0.05); it optimized long- and short-term glycemic control, with FBG, 2hPBG and HbA1c all reaching the ideal control range (P < 0.05); and it achieved a high nursing satisfaction rate of 95.35% (P = 0.024). Notably, the intervention achieved a high patient adherence rate of 93.02% during the 3-month intervention period, fully proving its good acceptability and feasibility in the older population. The significance of using the IMB model as the guiding framework of this study lies in three core aspects, which are exactly the key advantages that distinguish it from conventional non-guided nursing interventions: first, it overcomes the core defect of conventional T2DM nursing, which only focuses on single-dimensional disease knowledge delivery and ignores the cultivation of patients’ self-management motivation and practical behavioral skills, leading to the common “intention-behavior gap” (patients know but do not act), and the IMB model constructs a closed loop of health behavior change through the three interrelated core constructs of information input, motivation stimulation and behavioral skill training, effectively solving this long-term problem in chronic disease nursing; furthermore, it realizes targeted adaptation to the multi-dimensional health needs of older T2DM patients, as unlike single-dimensional intervention models that only focus on a single outcome, the IMB model can simultaneously address the interwoven problems of cognitive decline, negative emotions and poor self-management ability in older patients, achieving the synergistic improvement of psychological state, cognitive function and glycemic control through self-management behavior intervention rather than fragmented independent interventions, which perfectly matches the complex health needs of older chronic disease patients; finally, it provides a standardized, operable and reproducible framework for clinical geriatric diabetes nursing, as the IMB model-guided intervention has clear implementation steps, standardized intervention monitoring and adherence assessment systems, and high patient acceptability, which can be easily promoted and applied in clinical settings, effectively solving the problem of non-standardized nursing intervention for older T2DM patients and providing a unified theoretical and practical basis for clinical nursing work. These findings confirm that the IMB model-guided nursing intervention is an effective, comprehensive and feasible strategy for geriatric T2DM care, which can not only improve the disease management outcomes and quality of life of older T2DM patients, but also provide an evidence-based theoretical framework and operable clinical intervention protocol for the standardized development of geriatric chronic disease nursing.

## Data Availability

The raw data supporting the conclusions of this article will be made available by the authors, without undue reservation.
